# Improving Breast Cancer Screening Coverage in Primary Healthcare: A Quality-Improvement Study (2023-2025)

**DOI:** 10.7759/cureus.108674

**Published:** 2026-05-11

**Authors:** Salwa A AlAwadh, Razan Z AlShammari, Hawra A AlJohi, Rawan M Aseeri, Fadwa T AlOhali, Aram M Alghamdi, Nawal AlAhmari, Shakir Alharthi

**Affiliations:** 1 Department of Family Medicine, Armed Forces Hospital, Dhahran, SAU; 2 College of Medicine, King Faisal University, Al-Ahsa, SAU; 3 Department of Continuous Quality Improvement and Patient Safety, Armed Forces Hospital, Dhahran, SAU

**Keywords:** breast cancer, population-based screening, primary healthcare, quality improvement, screening mammography, workflow redesign

## Abstract

Background and aim: Organized breast cancer screening reduces mortality; however, coverage within our primary healthcare (PHC) network was substantially below benchmarks. Barriers included limited awareness, fragmented workflows, suboptimal use of electronic systems, and constrained diagnostic capacity. Screening delivery relied on opportunistic identification rather than systematic recall, resulting in missed opportunities, limited performance monitoring, and prolonged diagnostic pathways. This study aimed to increase breast cancer screening coverage among eligible women aged 40-69 years attending PHC clinics from 1.0% in August 2023 to at least 76% by September 2025 through: (1) systematic identification of eligible women using electronic health records (EHR)-based prompts and recall systems; (2) increased completion of screening mammograms; (3) timely diagnostic follow-up (≤14 days); and (4) minimal disruption to clinical workflow.

Methods: A quality-improvement (QI) initiative was conducted from August 2023 to October 2024 using the Institute for Healthcare Improvement Model for Improvement and sequential Plan-Do-Study-Act (PDSA) cycles. A multi-disciplinary team reviewed weekly and monthly reports tracking mammography requests, completions, Breast Imaging Reporting and Data System (BI-RADS) results, and waiting times. Although active implementation concluded in October 2024, outcomes were monitored through September 2025 to assess sustainability. Four PDSA cycles addressed as follows: (1) staff awareness and readiness, (2) structured patient recall using electronic health record-generated eligibility lists and outreach, (3) performance transparency via dashboards and feedback, and (4) workflow integration with periodic audits to support sustainability. The primary outcome was screening coverage. Secondary outcomes included BI-RADS distribution and confirmed malignancies. Balancing measures were mammography waiting time and physician perceptions.

Results: Documented screening coverage increased from 1.0% (n=49) in August 2023 to 70% (n≈3,400 of 4,857 eligible women) by September 2025. During the project period, a total of 1,652 screening mammograms were completed. Among completed mammograms, 68.2% (n=1,127) were BI-RADS 1-2, 18.9% (n=312) BI-RADS 0, 11.6% (n=192) BI-RADS 3, and 1.3% (n=21) BI-RADS 4-5, yielding 11 confirmed malignancies. Waiting times improved following capacity adjustments. Physician survey responses (n=36) indicated good acceptability and perceived feasibility within routine practice.

Conclusions: A multi-component, data-driven QI strategy integrated into PHC workflows was feasible and associated with substantial and sustained improvement in breast cancer screening coverage while maintaining timely diagnostic follow-up.

## Introduction

Breast cancer screening through mammography is a cornerstone of early detection and mortality reduction; however, screening coverage within the primary healthcare (PHC) network was substantially below benchmarks at project initiation. Baseline assessment in August 2023 demonstrated that only 1.0% (n=49) of eligible women aged 40-69 years (n=4,857) had a documented up-to-date screening mammogram. This represented a major gap in preventive care delivery.

Screening relied primarily on opportunistic identification rather than systematic recall, resulting in missed opportunities, inconsistent follow-up, and limited performance transparency. Fragmented workflows, absence of automated eligibility tracking, and constrained radiology capacity further limited structured delivery.

Breast cancer remains the most commonly diagnosed malignancy among women in Saudi Arabia and a leading contributor to cancer-related morbidity and mortality [1-3]. Organized mammography screening programs are associated with reductions in breast cancer mortality when delivered with adequate coverage, quality assurance, and timely diagnostic follow-up [4-7]. International guidance emphasizes that systematic recall-based screening models outperform opportunistic approaches [4,5,8,9].

Despite national preventive guidelines and free access to screening in Saudi Arabia [10-12], participation rates remain suboptimal across the Gulf Cooperation Council (GCC) countries [13-16]. Reported barriers include limited awareness, cultural concerns, fragmented referral pathways, absence of automated recall systems, and insufficient coordination between PHC and radiology services [17-20].

Evidence from randomized and quasi-experimental studies demonstrates that multi-component strategies integrating reminder and recall systems, provider prompts, audit and feedback, and EHR-enabled workflows produce greater and more sustained improvements in screening uptake than single-component educational interventions [21-29]. However, implementation-focused evidence describing comprehensive, workflow-integrated quality-improvement (QI) initiatives embedded within routine PHC settings in Saudi Arabia remains limited.

This initiative was guided by the Institute for Healthcare Improvement (IHI) Model for Improvement. The underlying assumption was that meaningful improvement in screening coverage would require coordinated system-level redesign. Embedding age-based eligibility prompts into routine consultations, implementing structured recall pathways, enhancing transparency through performance dashboards, and aligning radiology capacity with increased demand were expected to normalize screening within daily PHC workflows and reduce missed opportunities. These mechanisms are consistent with implementation evidence demonstrating improved delivery of preventive care when screening is integrated into standardized workflows supported by electronic health systems and audit-feedback processes [26-29].

## Materials and methods

Context

This QI project was conducted within the PHC network of the Armed Forces Hospital in Dhahran (AFHD), Saudi Arabia. The PHC service includes family medicine physicians, nurses, health educators, administrative staff, and coordinated radiology services. Eligible participants were women aged 40-69 years registered in PHC clinics without a documented screening mammogram within the preceding two years; women with a prior diagnosis of breast cancer were excluded.

During the project period, the PHC network transitioned from a legacy hospital information system (HIS) that required manual verification of screening status before patient discharge to a newer system that incorporated automated eligibility alerts and mandatory pop-up verification. This transition standardized documentation and strengthened workflow integration.

**Figure 1:**
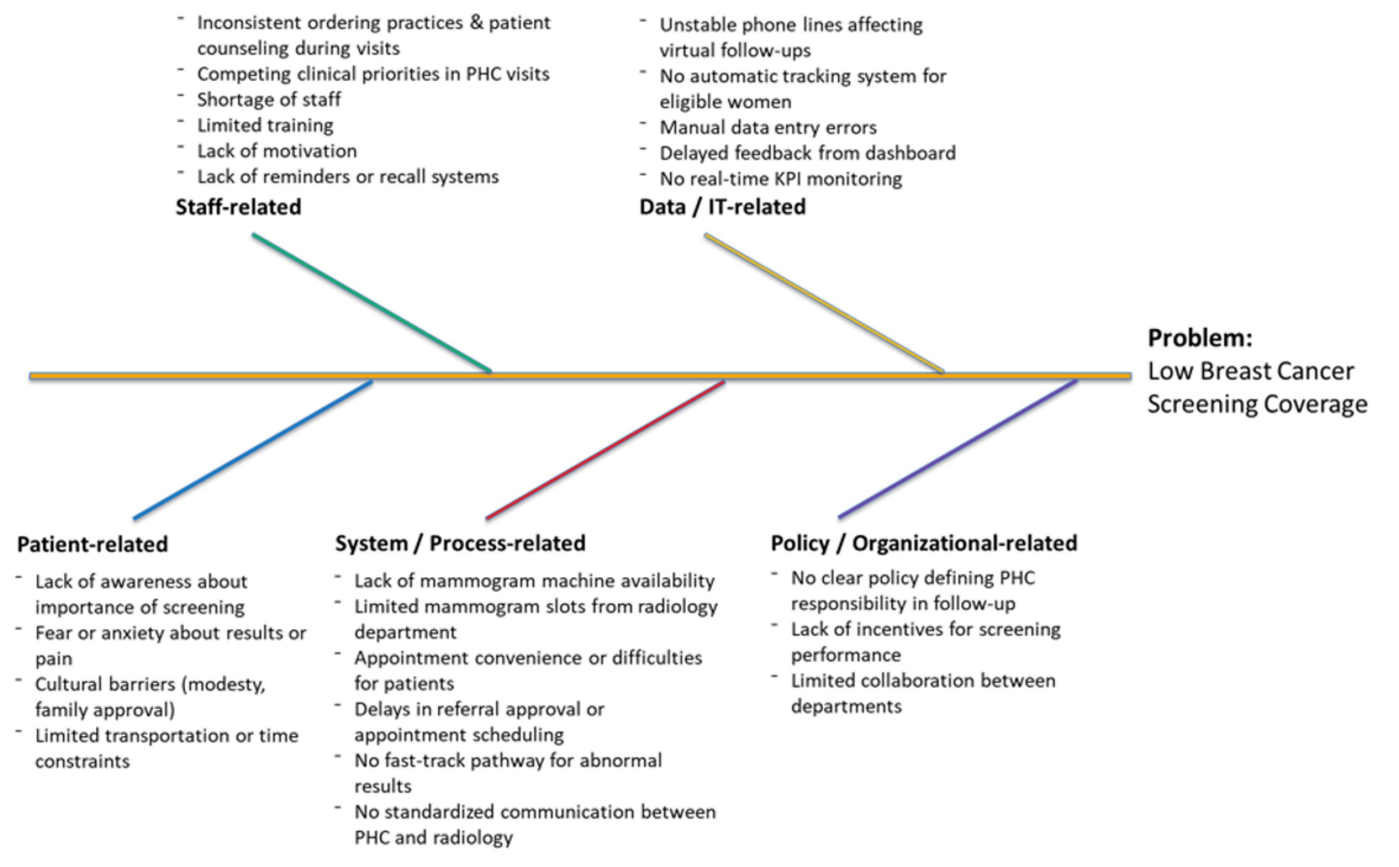
Fishbone diagram of factors contributing to low breast cancer screening coverage. PHC: primary healthcare

**Figure 2:**
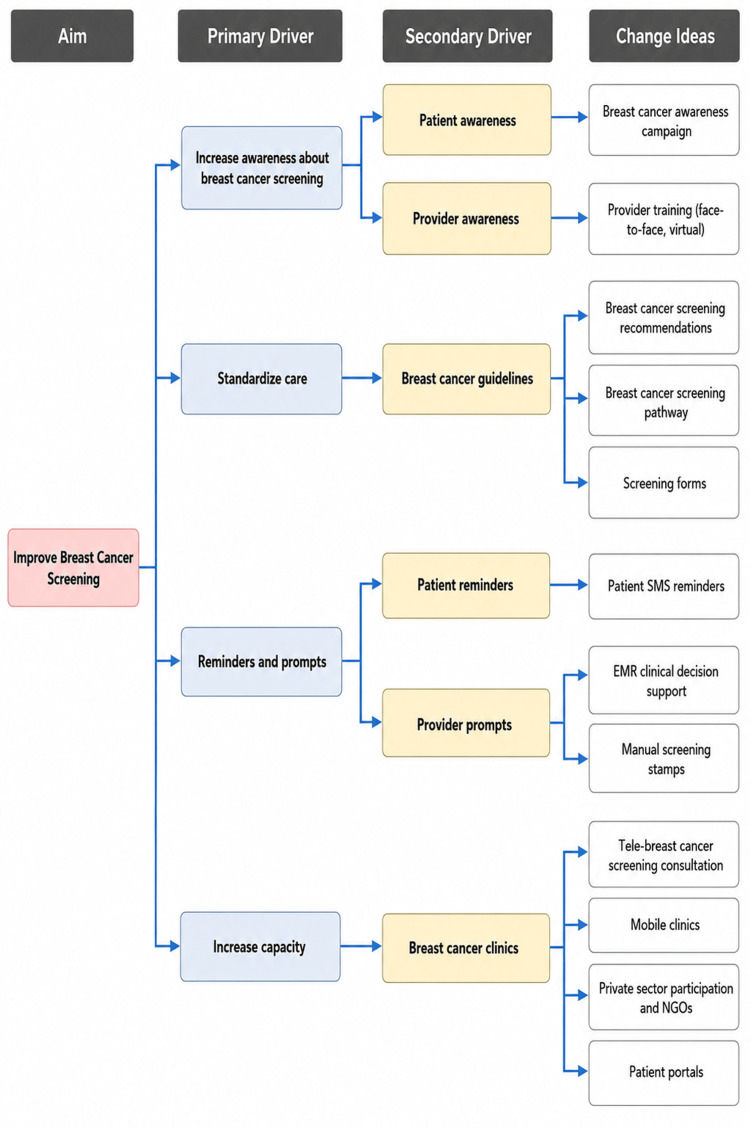
Driver diagram illustrating the QI strategy to increase breast cancer screening coverage in PHC. PHC: primary healthcare; QI: quality-improvement; NGO: non-governmental organization; EMR: electronic medical record

A root cause analysis using a fishbone diagram was conducted to identify key barriers to screening uptake, informed by baseline performance data, multi-disciplinary team discussions, and frontline workflow observations (Figure 1). A driver diagram was subsequently developed to link the project aim with primary and secondary drivers and proposed change ideas (Figure 2). The identified barriers were mapped to targeted interventions using a structured barrier-solution matrix (Table 1).

**Table 1:** Improvement area barrier-solution (countermeasures) matrix. PHC: primary healthcare; EHR: electronic health record; KPI: key performance indicators

Improvement area	Obstacles	Change ideas/interventions
Access and scheduling efficiency	Limited mammogram slots, delayed referral approval, and no fast-track for abnormal results	Coordination with radiology to increase slots, prioritization of overdue patients, and fast-track for abnormal results
Patient awareness and engagement	Lack of educational materials, no recall or reminder system, and cultural fear or misunderstanding	SMS recall messages, educational sessions, posters, and leaflets, community campaigns, counseling during chronic/well-woman visits, and referral of no-show patients to health education department for counseling follow-up, and rescheduling
Staff workflow and coordination	Inconsistent counseling, unclear role distribution, and lack of communication between PHC and radiology	Monthly KPI meetings, assigning a nurse navigator per clinic, orientation and reminder training for staff, and recognition of high-performing providers
Data tracking and feedback systems	Delayed or inaccurate KPI data, manual data entry errors, and no regular feedback to teams	Use of KPI dashboard, reminder activation, cross-verification with EHR data, and quarterly performance review and feedback

Potential change ideas were prioritized based on feasibility, expected impact, and alignment with identified barriers. Interventions with the highest feasibility and anticipated impact were selected for sequential testing through Plan-Do-Study-Act (PDSA) cycles.

Intervention

The intervention consisted of coordinated system-level changes targeting the identified barriers and was implemented from August 2023 to October 2024 using the Institute for Healthcare Improvement (IHI) Model for Improvement and sequential PDSA cycles. Program performance was subsequently monitored through September 2025 to assess the sustainability and stability of the improvements over time. A consolidated summary of the implemented interventions and system enhancements is provided in Table 2.

**Table 2:** Summary of implemented interventions and system enhancements. HIS: hospital information system; BI-RADS: Breast Imaging Reporting and Data System; EHR: electronic health record; KPI: key performance indicators

Intervention/action	Date/frequency of implementation
Educated patient and caregivers regarding breast cancer, screening guidelines, and target screening population	Monthly
Conducted community awareness campaigns for breast cancer screening	October 2023, 2024, and 2025
Monitored and optimized clinic workflow to improve efficiency and patient experience	Started August 2023
Implemented EHR alerts to notify physicians of screening eligibility and completion status	Started November 2023
Educated healthcare providers on the breast cancer screening system and workflow	Started August 2023
Trained the project team on data collection, analysis, and KPI reporting	Started August 2023
Expanded mammography service capacity through training and full utilization of a newly acquired mammography machine	Started August 2023
Implemented follow-up and rescheduling for patients who did not attend scheduled mammography appointments	Started April 2024
Delivered targeted awareness messages to eligible women for breast cancer screening	Started October 2024
Implemented virtual communication of results, including expedited referral for BI-RADS 4 and 5 findings	Started November 2023
Established weekly monitoring reports, including request dates, mammography dates, completed studies, no-shows, and BI-RADS outcomes	Started June 2024
Introduced physician performance feedback and recognition for high mammography referral rates	Started March 2024
Expedited procurement and installation of hospital mammography equipment	November 2023
Engaged specialized radiologists in mammography interpretation (part-time)	December 2023
Added a website-based option for booking virtual appointments for early breast cancer screening	October 2024
Transitioned to the new HIS with activation of automated mammography reminder alerts	August 2025

The QI initiative was led by a multi-disciplinary team including family physicians, a nurse navigator responsible for patient recall and follow-up, a radiology liaison coordinating appointment capacity, a data coordinator overseeing EHR extraction and KPI validation, a QI coordinator supporting dashboard monitoring, and IT personnel ensuring system functionality. Clear role delineation supported accountability and continuity throughout implementation. Four sequential PDSA cycles were conducted to iteratively test and refine interventions addressing barriers identified during root-cause analysis.

PDSA cycle one (August 2023-October 2023) focused on building awareness and operational readiness through provider education, patient awareness activities, workflow review, team training, and expansion of mammography service capacity. This cycle was piloted in selected clinics, with feedback used to refine awareness activities and workflow adjustments.

PDSA cycle 2 (November 2023-February 2024) introduced system-level changes, including EHR eligibility alerts, structured recall mechanisms, expedited referral pathways, and engagement of specialized radiologists to support increased screening demand. This cycle involved pilot testing of EHR alerts and recall processes, with iterative refinement based on usability and response rates.

PDSA cycle 3 (March 2024-July 2024) emphasized performance monitoring and accountability through physician performance feedback, follow-up of missed appointments, and implementation of weekly monitoring reports. Dashboards and reports were introduced incrementally and refined based on physician engagement and observed performance variation.

PDSA cycle 4 (August 2024-October 2024) focused on strengthening sustainability through enhanced patient outreach, targeted awareness messaging, and the introduction of a virtual appointment booking option for screening services. Interventions were embedded into routine workflows and refined based on utilization patterns to support sustainability. After completion of the active intervention phase, program performance was monitored through September 2025 to assess sustainability, including the transition to a new hospital information system with automated screening reminder alerts.

Study of the intervention

The impact of the intervention was evaluated through longitudinal monitoring of outcome, process, and balancing measures. Weekly and monthly performance reports were reviewed to assess temporal changes in screening requests, completions, BI-RADS distribution, and waiting times. Run charts and statistical process control (SPC) methods were used to examine variation and identify temporal associations between successive PDSA cycles and observed performance changes.

Measures

The primary outcome measure corresponding to the project aim was screening coverage, defined as the proportion of eligible women with a completed screening mammogram within the recommended interval. Secondary outcomes included BI-RADS distribution, number of biopsy-confirmed malignancies, and referral timeliness. Process measures assessed physician training completion and physician feedback regarding the usability of the EHR screening prompts during implementation. Balancing measures included mammography waiting time (completion within 14 days of request) and physician perceptions of workload and workflow integration, assessed through a structured post-intervention survey (five Likert-scale items). The survey instrument was developed by the study team based on workflow integration domains and was reviewed for face validity by senior clinicians.

Analysis

Descriptive statistics and time-series analyses were performed. SPC charts were used to assess process stability and detect special-cause variation. Data were extracted from the EHR and PHC KPI dashboards and cross-verified with radiology logs to ensure completeness and accuracy. Data extraction procedures were standardized using predefined EHR queries, and data completeness was verified through cross-checking with radiology logs.

Baseline screening coverage was measured in August 2023, prior to implementation of the improvement interventions. Initial implementation activities began during August-October 2023. Continuous monthly monitoring using standardized electronic reports began in November 2023, when automated data extraction from the EHR and PHC KPI dashboard became fully operational. Earlier baseline estimates were derived from retrospective review of clinical records.

Ethical considerations

This project was reviewed and approved by the Institutional Review Board of Armed Forces Hospitals Eastern Province, Saudi Arabia (IRB approval no: AFHER-IRB-2026-005), and was classified as a QI initiative. The patient-level component of the study utilized routinely collected, de-identified clinical and administrative data, and no additional interventions beyond standard care were introduced. Therefore, the requirement for individual patient informed consent was waived in accordance with institutional policy.

For the physician perception survey component, participation was voluntary. Physicians received an information sheet explaining the purpose of the survey, and informed consent was obtained prior to participation. Survey responses were collected anonymously and analyzed in aggregate form.

All data used for analysis were de-identified and reported only in aggregate form. Data were stored on password-protected institutional systems with access restricted to authorized study personnel, in accordance with institutional data protection and confidentiality policies. Patients and members of the public were not involved in the design, conduct, reporting, or dissemination plans for this quality-improvement initiative. This manuscript was prepared in accordance with the Standards for Quality Improvement Reporting Excellence (SQuIRE) 2.0 reporting guidelines for healthcare improvement studies.

## Results

Data were collected from August 2023 to September 2025 across the PHC network at Armed Forces Hospital in Dhahran (AFHD). A total of 1,652 mammogram reports were analyzed, covering BI-RADS categories 0-5. The dataset included monthly records of mammogram requests and completed procedures, patient waiting times, physician training attendance, and clinic visit volumes.

Outcome measures

Breast Cancer Screening Coverage

The screening initiative demonstrated a marked and sustained improvement in documented breast cancer screening coverage among eligible women. At baseline in August 2023, screening coverage was 1.0% (n=49) among eligible women (n=4,857), reflecting a very limited number of women with documented mammograms within the PHC system. A small number of these baseline screenings had been performed outside the facility and were recorded retrospectively rather than through the organized PHC screening workflow.

**Figure 3:**
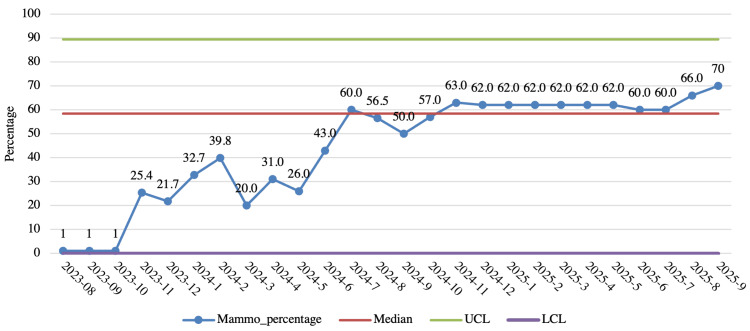
Monthly breast cancer screening coverage among eligible women during the QI initiative. QI: quality-improvement; UCL: upper control limit; LCL: lower control limit

Following implementation of the QI interventions, screening coverage increased progressively, reaching 70% (n≈3,400 of 4,857 eligible women) by September 2025, demonstrating a clear upward trend over the observation period (Figure 3). During the project period, a total of 1,652 screening mammograms were completed, reflecting the substantial increase in screening activity generated by the intervention.

It is important to note that screening coverage represents the proportion of eligible women with a documented up-to-date mammogram within the recommended interval, and therefore exceeds the number of mammograms performed during the project period alone. Coverage improved steadily after the introduction of the interventions and approached the institutional benchmark target of 76% by the end of the study period.

Screening Mammogram Findings

**Figure 4:**
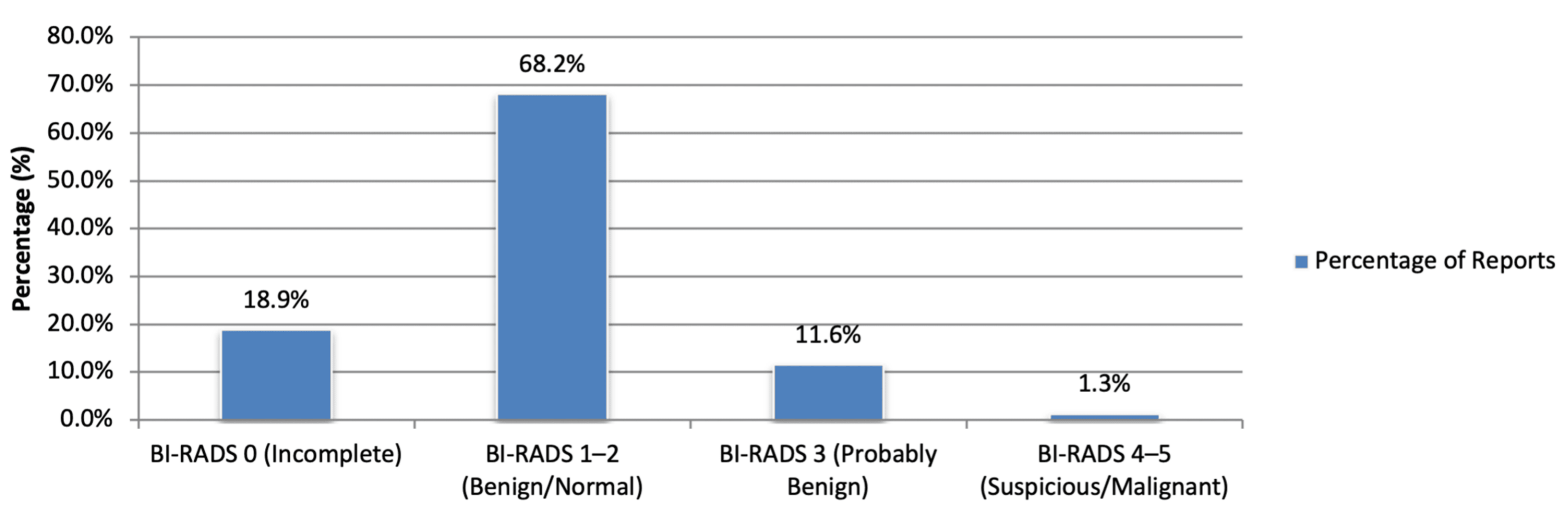
Distribution of BI-RADS categories among completed screening mammograms. BI-RADS: Breast Imaging Reporting and Data System

Among the (n=1,652) mammogram reports, the distribution of Breast Imaging Reporting and Data System (BI-RADS) categories is presented in Figure 4. The majority of examinations (n=1,127; 68.2%) were classified as BI-RADS 1-2, indicating normal or benign findings. BI-RADS 0 accounted for 18.9% (n=312), reflecting cases requiring additional imaging, while BI-RADS 3 represented 11.6% (n=192) of reports. BI-RADS 4-5, indicating suspicious findings suggestive of malignancy, accounted for 1.3% (n=21) of examinations. Subsequent diagnostic follow-up confirmed (n=11) malignancies, each verified by histopathology and referred to specialized oncology services for further management. The median referral-to-consultation interval was 14 days, indicating timely coordination of diagnostic and treatment pathways.

Process Measures

Process indicators were monitored to assess implementation fidelity and operational performance. Physician training on breast cancer screening and referral processes improved from 90% in November 2023 to 100% by January 2024 and remained at full compliance through September 2025.

**Figure 5:**
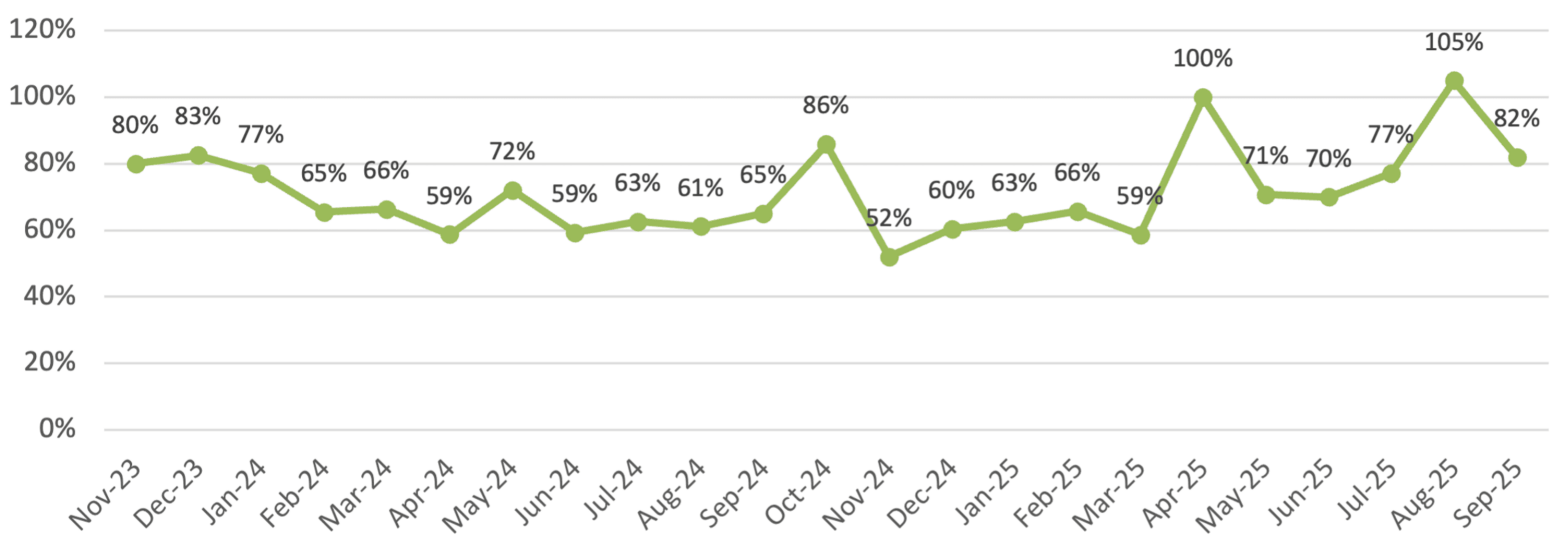
Monthly completion rate of mammogram requests (2023-2025).

During the implementation phase, physicians also provided ongoing feedback regarding their satisfaction with the usability of the EHR screening prompts. Informal monitoring indicated that the prompts were generally perceived as helpful in identifying eligible patients during routine clinical encounters. In addition, the monthly completion rate of requested mammograms improved progressively over the study period, reflecting improved coordination between PHC clinics and radiology services (Figure 5).

Balancing measures

Waiting Time for Screening Mammography

**Figure 6:**
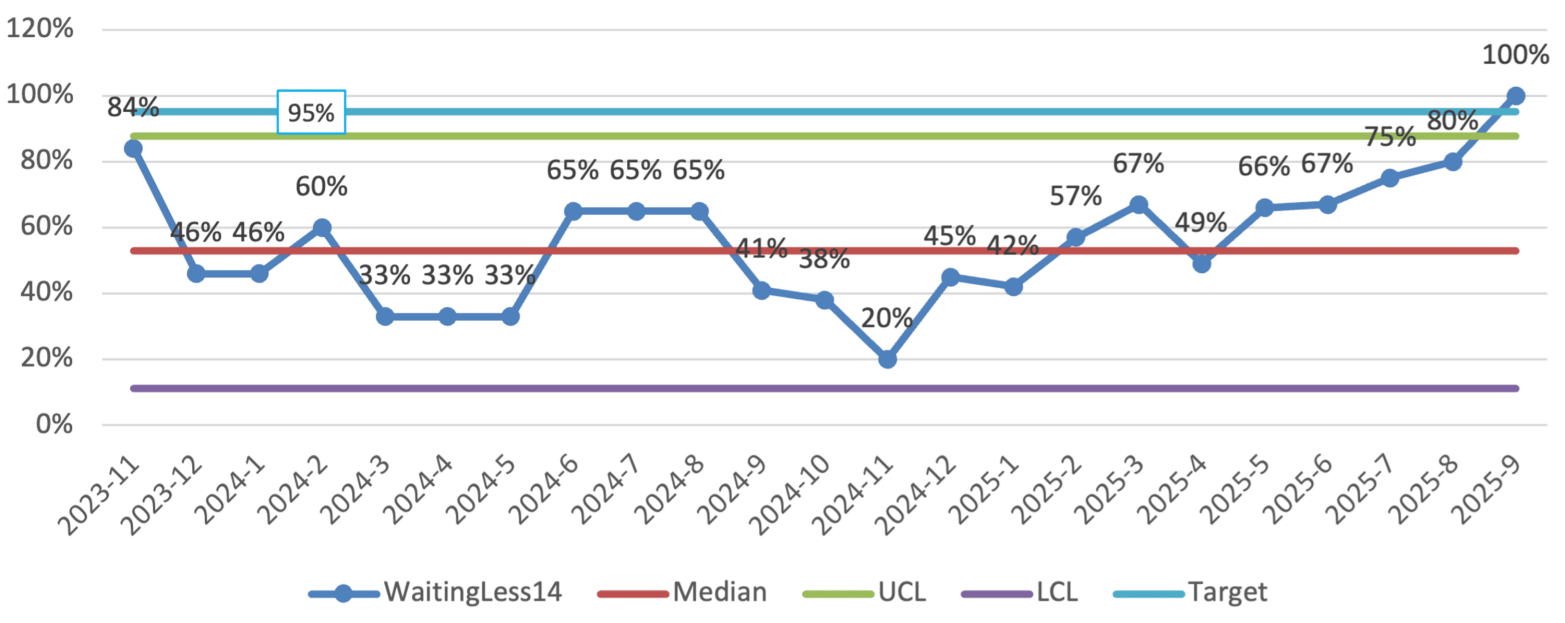
Percentage of patients completing mammogram within 14 days (2023-2025). UCL: upper control limit; LCL: lower control limit

Waiting time for screening mammography was evaluated as a balancing measure. The proportion of patients completing mammography within 14 days of request fluctuated during the observation period (Figure 6). Performance declined temporarily following the initial increase in screening demand, but improved gradually after the implementation of capacity-building measures, such as additional radiology slots and scheduling adjustments.

Physician Perception and Workload Assessment

Following completion of the intervention, a physician perception survey (n=36) was conducted to assess the impact of the initiative on clinical workflow and preventive care delivery. Most physicians reported that the screening process was well integrated into their workflow (n=29; 80.5%), while only a minority perceived an increase in workload (n=7; 19.4%) or interference with other preventive services (n=4; 11.1%) (Table 3).

**Table 3:** Summary of physician survey findings on balancing measures (n=36). EHR: electronic health records; HIS: hospital information system; QI: quality-improvement

Domain/statement (abbreviated)	Mean score	Agree or strongly agree (%)
QI interventions increased my daily workload	3.39	19.4
EHR documentation time reasonable (legacy HIS)	4.11	55.6
EHR documentation time reasonable (new HIS)	4.83	91.6
Interventions affected ability to manage other preventive services	3.22	11.1
Screening process well integrated into my workflow	4.61	80.5

## Discussion

This QI project aimed to increase documented breast cancer screening coverage among eligible women attending the PHC network by strengthening awareness, recall systems, EHR prompts, and radiology capacity. At baseline in August 2023, only 1.0% (n=49) of eligible women (n=4,857) had a documented up-to-date screening mammogram. By September 2025, screening coverage had increased to 70% (n≈3,400 of 4,857 eligible women). The BI-RADS distribution remained within expected screening ranges, with most reports classified as BI-RADS 1-2 (n=1,127; 68.2%), 18.9% (n=312) as BI-RADS 0, 11.6 (n=192) as BI-RADS 3, and 1.3% (n=21) as BI-RADS 4-5. Eleven biopsy-confirmed malignancies were identified and referred, with a median referral time of 14 days from diagnosis to surgical or oncology clinics. These findings indicate that the intervention bundle achieved the specific aim of markedly improving breast cancer screening coverage while maintaining diagnostic performance and timeliness.

The pattern of improvement across outcome and process measures, aligned with the timing of the PDSA cycles, indicates a strong association between the intervention bundle and the observed gains. Early increases in ordering and completion rates followed provider education, awareness efforts, and the introduction of age-based EHR prompts, which made eligibility visible during consultations. Coverage accelerated further with the use of daily eligibility lists, standardized recall scripts, and SMS reminders, supporting more consistent identification and follow-up of eligible women. Dashboards, feedback, and the monthly champion model helped sustain provider engagement. Radiology capacity enhancements and the transition to new HIS-enabled higher throughput without extending referral intervals for BI-RADS 4 and 5 cases. While causality cannot be confirmed due to the observational design, the temporal sequence, internal consistency of results, and concordance with established mechanisms described in the literature suggest that the interventions were likely associated with the observed improvements.

The scale of improvement in this project is consistent with, and in some respects exceeds, effects reported in the literature for similar implementation strategies. International evidence from randomized and quasi-experimental studies shows that client reminders, including letters, telephone calls, and SMS messages, typically increase screening participation by about 5-20% [26-29]. In a recent US trial, behavioral nudges that combined bulk ordering with text messaging significantly increased mammography uptake compared with usual care [26], and other work confirms that multiple SMS reminder interventions can increase scheduling and completion rates [27]. Provider-focused interventions, such as EHR alerts, standing orders, and audit with feedback, have also been associated with meaningful increases in cancer screening rates and improved follow-through on overdue preventive care [28-31]. These findings are consistent with the improvements seen in our project after the introduction of age-based EHR prompts, structured recall, and regular performance feedback.

Similarly, a recent QI study from Saudi Arabia that applied the Find, Organize, Clarify, Understand, Select - Plan, Do, Check, Act (FOCUS-PDCA) methodology demonstrated that reorganizing breast imaging workflows, strengthening physician engagement, and standardizing referral pathways substantially increased mammography screening activity and improved reporting quality in a tertiary hospital setting [32]. Although conducted in a Saudi PHC setting, the intervention components are applicable to other systems, including the United States, where similar strategies have improved screening uptake [26-29]. Comparable barriers, such as missed opportunities, fragmented care, and patient-level factors, support the transferability of this approach with local adaptation.

In the regional context, studies from Saudi Arabia and other Gulf Cooperation Council (GCC) countries consistently report low baseline participation in organized mammography programs, with uptake often in the range of 1-10% despite free access [13-17]. Recent national and subnational evaluations highlight persistent gaps in awareness, cultural hesitancy, access, and system-level organization [18-20]. Scoping reviews and empirical studies from the Gulf region emphasize that culturally tailored education, Arabic-language messaging, and community-based campaigns can improve knowledge and intention to screen, but that translating these into sustained participation requires stronger system integration and reliable recall mechanisms [33-35]. The coverage achieved in the present project, reaching 70%, compares favorably with these reports and approaches participation levels recommended by the WHO Global Breast Cancer Initiative (GBCI), which targets at least 60% of cancers detected at an early stage [8,9]. The distribution of BI-RADS findings in our cohort is also comparable to benchmarks reported from large screening programs internationally, where most examinations are reported as negative or benign and a small proportion, typically around 1-2%, fall into BI-RADS 4 and 5 categories [36,37].

The intervention bundle had several important effects on patients, providers, and the health system. For patients, the project improved access to timely screening through clearer pathways, proactive recall, and the availability of virtual booking options. This likely reduced missed opportunities among women who might otherwise not have been offered screening during routine visits, a phenomenon described in other systems where screening relies solely on opportunistic offers [37]. For providers, the introduction of EHR prompts, standardized workflows, and feedback reports helped integrate screening into daily practice and reduced reliance on memory or individual initiative. Similar to reports from other EHR-based interventions, clinicians in our project reported that prompts and standardized orders made it easier to act on screening recommendations and coordinate with radiology services [28,30]. At the system level, the project improved data visibility across PHC and radiology, strengthened communication channels, and supported more efficient use of the mammography machine through protected slots and coordinated scheduling. These system changes are aligned with broader international efforts to embed breast cancer screening within organized programs rather than fragmented opportunistic approaches [4,17].

Despite substantial improvement, the project did not fully reach the national benchmark of 76% screening coverage by the end of the observation period. Several contextual factors likely contributed to this gap. First, demand increased more rapidly than radiology capacity in some phases, resulting in fluctuating waiting times and periodic backlogs. Similar capacity constraints and variability in waiting times have been documented in other screening programs undergoing rapid scale-up [37]. Second, the transition to the new HIS, while beneficial in the long term, introduced short-term disruption as staff adjusted to new workflows and to dual-system use. Third, persistent cultural and psychosocial barriers, including fear of diagnosis, embarrassment, and perceptions of low personal risk, remain common among Saudi women and may have limited participation among some eligible individuals even when outreach and reminders were in place [18,20,34]. Finally, differences in engagement between clinics, influenced by local leadership, staffing, and competing priorities, produced variation in performance and may have slowed aggregate progress. These contextual influences underscore the importance of continuous adaptation, local ownership, and ongoing support when implementing complex system-level change.

This QI project generated several important lessons regarding the implementation of organized breast cancer screening within PHC. First, provider engagement supported by clear EHR prompts was critical to improving consistency in screening orders. Embedding age-based eligibility prompts into routine consultations reduced reliance on individual clinician recall and normalized screening as part of everyday practice. Ongoing education and feedback reinforced adherence to the pathway and helped sustain engagement over time. Second, structured patient outreach using daily eligibility lists, standardized recall scripts, and SMS reminders substantially improved follow-up and completion rates. Repeated, culturally appropriate communication was particularly important in addressing hesitation and missed opportunities, demonstrating that single-contact approaches were insufficient for sustained uptake. Third, the project highlighted the need to align demand generation with diagnostic capacity. Periods of rapid improvement placed pressure on appointment availability, emphasizing that outreach strategies must be accompanied by proactive radiology capacity planning to avoid bottlenecks. Fourth, system transitions introduced both challenges and learning opportunities. The shift to a new EHR platform temporarily disrupted workflows and data capture, requiring additional staff support, workflow redesign, and close monitoring. This experience underscored the importance of structured onboarding, clear role delineation, and iterative adjustment when implementing digital system changes. Finally, sustained collaboration among PHC, radiology, and IT teams was essential for timely problem-solving and continuity, reinforcing that complex system-level improvements require shared ownership rather than isolated interventions.

Several limitations should be considered. The project was conducted within a single institutional PHC network, which may limit generalizability to other settings with different populations, resources, or governance structures. The before-and-after observational design without a concurrent control group restricts causal inference and does not fully account for secular trends, external awareness campaigns, or policy changes. Measurement limitations included early documentation gaps, particularly for mammograms performed outside the facility, which may have resulted in an underestimate of baseline coverage. Temporary inconsistencies in data entry during the EHR transition may have led to misclassification despite cross-verification using multiple data sources. Estimates of waiting time depended on accurate recording of request and appointment dates and may have been influenced by patient-initiated rescheduling. In addition, the transition to a new HIS during the study period may have independently influenced screening documentation and workflow efficiency, representing a potential confounding factor. Furthermore, screening coverage may partially reflect previously completed or externally performed mammograms that were retrospectively documented, potentially overestimating the direct effect of the intervention. Finally, patient-centered outcomes such as patient satisfaction, perceived barriers, and stage at diagnosis were not systematically assessed and should be addressed in future work.

Provider perception data were subject to response and social desirability bias, and the modest response size may not reflect all viewpoints. In addition, patient-level outcomes such as stage at diagnosis and patient experience were not assessed. Efforts to minimize these limitations included time-series monitoring, triangulation of data sources, and iterative refinement of measurement processes; however, residual confounding cannot be excluded. Future cycles would benefit from longer follow-up, additional patient-reported outcomes, and multi-site implementation to strengthen external validity.

## Conclusions

This QI project achieved its stated aim of substantially improving breast cancer screening coverage within PHC while maintaining diagnostic quality and referral timeliness. Screening coverage increased from 1% to 70% over the project period, with sustained improvement observed beyond the active implementation phase. The use of outcome, process, and balancing measures allowed continuous assessment of performance, workload, and unintended consequences, supporting adaptive decision-making throughout the project. The intervention was feasible within existing resources and demonstrated that integrating EHR-based prompts, structured recall systems, patient outreach, and coordinated radiology capacity can produce meaningful and durable improvements. While no formal cost analysis was conducted, the project leveraged existing infrastructure and staff roles, suggesting potential efficiency gains through reduced missed opportunities and improved coordination rather than additional financial investment.

The improvements appear sustainable, supported by embedded workflows, routine performance monitoring, and continued stakeholder engagement. The approach is transferable to other PHC settings, provided that recall pathways, communication strategies, and capacity planning are adapted to the local context. Future work should explore predictors of non-participation, patient experience, linkage with cancer registry data, and evaluation across multiple sites using more rigorous comparative designs to guide wider scale-up and policy implementation.
